# Evaluation of Reference Genes for Quantitative Real-Time PCR in Oil Palm Elite Planting Materials Propagated by Tissue Culture

**DOI:** 10.1371/journal.pone.0099774

**Published:** 2014-06-13

**Authors:** Pek-Lan Chan, Ray J. Rose, Abdul Munir Abdul Murad, Zamri Zainal, Eng-Ti Leslie Low, Leslie Cheng-Li Ooi, Siew-Eng Ooi, Suzaini Yahya, Rajinder Singh

**Affiliations:** 1 Advanced Biotechnology and Breeding Centre, Malaysian Palm Oil Board (MPOB), No. 6, Persiaran Institusi, Bandar Baru Bangi, Kajang, Selangor, Malaysia; 2 Australian Research Council Centre of Excellence for Integrative Legume Research, School of Environmental and Life Sciences, The University of Newcastle, New South Wales, Australia; 3 School of Biosciences and Biotechnology, Faculty of Science and Technology, Universiti Kebangsaan Malaysia, UKM Bangi, Selangor, Malaysia; 4 Sime Darby Biotech Laboratories Sdn Bhd, Km10, Jalan Banting-Kelanang, Banting, Selangor, Malaysia; ISA, Portugal

## Abstract

**Background:**

The somatic embryogenesis tissue culture process has been utilized to propagate high yielding oil palm. Due to the low callogenesis and embryogenesis rates, molecular studies were initiated to identify genes regulating the process, and their expression levels are usually quantified using reverse transcription quantitative real-time PCR (RT-qPCR). With the recent release of oil palm genome sequences, it is crucial to establish a proper strategy for gene analysis using RT-qPCR. Selection of the most suitable reference genes should be performed for accurate quantification of gene expression levels.

**Results:**

In this study, eight candidate reference genes selected from cDNA microarray study and literature review were evaluated comprehensively across 26 tissue culture samples using RT-qPCR. These samples were collected from two tissue culture lines and media treatments, which consisted of leaf explants cultures, callus and embryoids from consecutive developmental stages. Three statistical algorithms (geNorm, NormFinder and BestKeeper) confirmed that the expression stability of novel reference genes (*pOP-EA01332*, *PD00380* and *PD00569*) outperformed classical housekeeping genes (*GAPDH*, *NAD5*, *TUBULIN*, *UBIQUITIN* and *ACTIN*). *PD00380* and *PD00569* were identified as the most stably expressed genes in total samples, MA2 and MA8 tissue culture lines. Their applicability to validate the expression profiles of a putative ethylene-responsive transcription factor 3-like gene demonstrated the importance of using the geometric mean of two genes for normalization.

**Conclusions:**

Systematic selection of the most stably expressed reference genes for RT-qPCR was established in oil palm tissue culture samples. *PD00380* and *PD00569* were selected for accurate and reliable normalization of gene expression data from RT-qPCR. These data will be valuable to the research associated with the tissue culture process. Also, the method described here will facilitate the selection of appropriate reference genes in other oil palm tissues and in the expression profiling of genes relating to yield, biotic and abiotic stresses.

## Introduction

Oil palm (*Elaeis guineensis*), which originated from West Africa, is a diploid monocotyledon that belongs to the Arecaceae family and Elaeidinae sub-tribe [1]. It is one of the most economically important plantation crops in Malaysia and accounts for 5% of the world vegetable oils cultivation area [2]. Being the highest yielding oil crop, oil palm produces up to 10 times more oil per hectare of land compared to other major oil cropsError! Hyperlink reference not valid [3]. Two types of oil can be extracted from oil palm fruits. Oil from the mesocarp is known as palm oil and is used mainly in the food based industry, while palm kernel oil from the endosperm is essential for the oleochemical industry. In addition, palm oil can be converted into biodiesel [4].

With the emergence of next generation sequencing technology, the availability of genome information has now expanded for oil palm. The first 1.8-gigabase genome sequence of African oil palm *Elaeis guineensis* with at least 34,802 genes has recently been published by Singh et al. [2]. Transcriptome sequences from oil palm tissues such as mesocarp, fruit, flower, endosperm and embryo have been deposited in the GenBank database [5], [6], [7]. An increasing number of candidate genes regulating complex traits in oil palm such as yield or disease resistance can now be investigated.

Increasing attention is being given to improve the stagnating yield of oil palm. The national average palm oil yield in Malaysia has plateaued at about 3.5 to 3.9 tonnes/hectare/year for more than two decades [8]. One approach to increased yield is the cultivation of superior planting material with high-yielding potential on the existing cultivated land. In order to expedite the production of palms with superior characteristics, clonal propagation using tissue culture has been identified since the 1970s as one of the promising tools. This process has been utilized widely in the oil palm industry to multiply elite planting materials [9], [10]. Results from the first oil palm clonal trials showed an increase of up to 30% in oil yield relative to the *dura* x *pisifera* palms planted from seeds [11].

However, the challenge encountered by tissue culturists is the low efficiency of the process itself. The callogenesis rate of leaf explants is around 19% [12], while the average rate for embryogenesis in leaf-derived callus is in the range of 3 to 6% [13]. Therefore molecular research is extensively carried out to understand the mechanisms underlying somatic embryogenesis (SE) in oil palm. This resulted in the identification of SE associated genes such as *EgLSD* (Lignostilbene-a,b-dioxygenase), *EgER6* (Ethylene responsive 6), *Eg*707 (unknown protein) and *EgIAA9* (putative member of the AUX/IAA gene family) [14], [15], [16]. There is increased opportunity to discover key SE regulatory genes with the availability of genome information. The most common and powerful technique used to explore the expression profiles of genes of interest (GOI) is reverse transcription quantitative real-time PCR (RT-qPCR), which is highly specific, sensitive and cost-effective [17], [18].

Accurate quantification of gene expression levels using RT-qPCR is highly dependent on the normalization of GOI with the most suitable reference genes. Recent developments have shown that more than one reference gene is required for optimum normalization of non-biological sample-to-sample variation introduced during RT-qPCR [19], [20]. As a result, the number of publications describing systematic evaluation of multiple reference genes in model and non-model plants has increased markedly, for example Arabidopsis [21], [22], rice [23], [24], soybean [25], banana [26], citrus [27] and bamboo [28].

Similar to other plant species, RT-qPCR studies in oil palm utilized the classical housekeeping genes such as *ACTIN* [14] and glyceraldehyde-3-phosphate dehydrogenase (*GAPDH*) [15] for normalization. As there is increasing evidence that these genes are not consistently expressed in certain plant species or experimental conditions [20], [29], [30], other genes have been investigated on their potential as reference genes. This resulted in the application of gibberellin-responsive protein 2 (*GRAS*), cyclophilin 2 (*CYP2*) and pre-mRNA splicing factor 7 (*SLU7*) as reference genes in the study of oil palm leaf discs subjected to various abiotic stresses [31]. These genes also showed the most stable expression across reproductive and vegetative tissues of oil palm [32]. In relation to SE in oil palm, the availability of expressed sequence tags (ESTs) [33], [34], [35], [36] and cDNA microarray expression data [37] have provided candidate reference genes for RT-qPCR.

Given the significance of the tissue culture process to the oil palm industry, expression stability of eight candidate reference genes suggested in preliminary studies by Low [37] and Ooi et al. [16] [predicted 40S ribosomal protein S27-2 (*PD00380*), manganese superoxide dismutase (*PD00569*), predicted protein IFH-1 like (*pOP-EA01332*), *GAPDH*, NADH dehydrogenase subunit 5-like gene (*NAD5*), alpha-tubulin 1 (*TUBULIN*), polyubiquitin (*UBIQUITIN*) and actin (*ACTIN*)] were investigated in this study across samples collected from various consecutive developmental stages of oil palm tissue culture, with cultured leaf explants sampled at different days, callus and embryoids (EMB). Different tissue culture lines and media treatments were also used. Detailed and systematic analyses were carried out using geNorm [19], NormFinder [38] and BestKeeper [39]. As a result of this comprehensive evaluation, two novel genes (*PD00380* and *PD00569*) were selected as the most stably expressed genes compared to classical housekeeping genes. Application of these genes to normalize the expression levels of an ethylene-responsive transcription factor 3-like gene (*PD00088)* in oil palm is also discussed.

## Results

### Candidate Reference Genes for Oil Palm Tissue Culture

A total of eight candidate genes were selected for determination of the most stable reference genes across various developmental stages of oil palm tissue culture. These samples which constituted leaf explant cultures, callus and EMB from consecutive developmental stages were collected from two different tissue culture lines (MA2, MA8) and media treatments (T527, T694). The selected genes were novel reference genes or classical housekeeping genes. As shown in Table 1, the novel reference genes selected and their GenBank accession number are *PD00380* (EY397675), *PD00569* (EL682210) and *pOP-EA01332* (EY406625), which were identified from an oil palm cDNA microarray study across embryogenic callus (EC), non-embryogenic callus (NEC), EMB, shoot from polyembryoids (ST), female inflorescence (INF), kernel at 12 weeks after anthesis (WAA), mesocarp at 15 WAA and roots from six months old seedling palms [37]. Microarray data from these samples were filtered for non-differentially expressed genes with the cutoff expression levels below 1.5 fold. Genes with missing data points were removed and the remaining genes were then ranked according to their standard deviation (SD). The novel reference genes selected for this study were among the top 75 genes with the lowest SD (File S1). Expression levels of these three novel reference genes were evaluated using RT-qPCR across the same samples as the microarray study with the addition of seven-day tissue culture explants and spear leaf (LEAF). GeNorm analysis showed that all the three genes were stably expressed in the tested tissue culture materials and mature tissues (File S2). Another five genes were classical housekeeping genes and their GenBank accession number are *GAPDH* (DQ267444), *NAD5* (DQ872924), *TUBULIN* (EL685625), *UBIQUITIN* (EL689143) and *ACTIN* (AY550991), which were selected based on a literature review [16].

**Table 1:** Candidate reference genes for evaluation across oil palm tissue culture samples.

Gene abbreviation	Gene name	GenBank accession number	Forward primer sequences (5′-3′)	Reverse primer sequences (5′-3′)	Amplicon length (bp)	Annealing temperature (°C)
*PD00380*	Predicted 40S ribosomal protein S27-2	EY397675	GATGGTTCTTCCGAACGATATTGA	TCACATCCATGAAGAATGAGTTCG	113	63
*PD00569*	Manganese superoxide dismutase	EL682210	CACCACCAGACGTACATCACAAA	GATATGACCTCCGCCATTGAACT	129	60
*pOP-EA01332*	Predicted protein IFH-1 like	EY406625	AAACGAAGGTACGGCAAGTACAAG	CTTAGCACATGCAGAGCAGATGTT	111	60
*GAPDH*	Glyceraldehyde 3-phosphate dehydrogenase	DQ267444	GATCGAGAAATCAGCCACGTATG	GTCACCAATAAAGTCGGTGGACA	124	60
*NAD5*	NADH dehydrogenase subunit 5-like gene	DQ872924	CATTTCTGGTTCACACGACTTCAG	AGAGAGTAAAACGACCCGAAATCC	112	60
*TUBULIN*	Alpha-tubulin 1	EL685625	CATGGCTTGCTGCCTTATGTATC	AGGACACCAGTCAACAAACTGGA	109	60
*UBIQUITIN*	Polyubiquitin	EL689143	CCAGGCCAATCTCTCAGGATG	GGGGGATGCCCTCTTTATCC	130	63
*ACTIN*	Actin	AY550991	TGCTGATCGTATGAGCAAGGAAA	GAAATCCACATCTGCTGGAAGGT	147	60

The biological role of the three novel reference genes has not been extensively studied. Classification of these genes together with well characterized classical reference genes provides a clearer idea regarding their putative function in the oil palm. Thus, functional annotation of the reference genes was performed using Blast2GO [40]. At level 2, the selected reference genes were assigned to various Gene Ontology (GO) terms associated with the three main ontologies, which are biological process, cellular component and molecular function. This analysis showed that the candidate genes were spread across different functional classes except for *pOP-EA01332*, which was only associated to three GO terms (cellular process, metabolic process and cellular component organization or biogenesis) under the biological process. The majority of the genes are involved in cellular and metabolic processes, in cell or organelle components and engaged in binding or catalytic activities (Table 2).

**Table 2:** Gene ontology classification of oil palm candidate reference genes at level 2 using Blast2GO.

GO classification	Number of sequences	Candidate reference genes
Biological Process		
Cellular process	8	*PD00380, PD00569, pOP-EA01332, ACTIN, TUBULIN, GAPDH, UBIQUITIN, NAD5*
Metabolic process	7	*PD00380, PD00569, pOP-EA01332, TUBULIN, GAPDH, UBIQUITIN, NAD5*
Response to stimulus	3	*PD00569, ACTIN, GAPDH*
Cellular component organization or biogenesis	3	*PD00380, pOP-EA01332, TUBULIN*
Developmental process	3	*PD00569, ACTIN, GAPDH*
Multicellular organismal process	3	*PD00569, ACTIN, GAPDH*
Reproduction	2	*PD00569, GAPDH*
Multi-organism process	2	*PD00569, GAPDH*
Growth	1	*ACTIN*
		
Cellular Component		
Organelle	7	*PD00380, PD00569, ACTIN, TUBULIN, GAPDH, UBIQUITIN, NAD5*
Cell	7	*PD00380, PD00569, ACTIN, TUBULIN, GAPDH, UBIQUITIN, NAD5*
Membrane-enclosed lumen	4	*PD00380, PD00569, ACTIN, GAPDH*
Membrane	4	*PD00380, ACTIN, GAPDH, NAD5*
Macromolecular complex	2	*PD00380, TUBULIN*
Symplast	1	*PD00380*
Cell junction	1	*PD00380*
Extracellular region	1	*GAPDH*
		
Molecular Function		
Binding	5	*PD00380, PD00569, ACTIN, TUBULIN, GAPDH*
Catalytic activity	5	*PD00569, TUBULIN, GAPDH, UBIQUITIN, NAD5*
Structural molecule activity	2	*PD00380, TUBULIN*
Antioxidant activity	1	*PD00569*

### PCR Amplification Efficiencies of Primer Pairs

For each of the candidate reference genes, a standard curve was generated across each tissue culture line with different media treatments (Figure S1). The estimated PCR amplification efficiencies (Ex) of these genes ranged from 81 to 104% (Table 3). The majority of the candidate reference genes exhibited average amplification efficiencies of more than 86% with the exception of *ACTIN*. Furthermore, the observed correlation coefficient (*R^2^*) values for most of the genes were greater than 0.99, which signified a strong correlation between the cycle threshold (Ct) values and the amount of cDNA template used in the amplification reactions.

**Table 3:** PCR amplification efficiencies (Ex) and correlation coefficient (*R* ^2^) values of oil palm candidate reference genes.

Gene abbreviation	Gene description	PCR amplification efficiencies, Ex (%)	Correlation coefficient *(R* ^2^)
		MA2 tissue culture line MA8 tissue culture line	MA2 tissue culture line MA8 tissue culture line
		MA2T527 MA2T694 MA8T527 MA8T694	MA2T527 MA2T694 MA8T527 MA8T694
*PD00380*	Predicted 40S ribosomal protein S27-2	89 86 88 93	0.9987 0.9856 0.9976 0.9977
*PD00569*	Manganese superoxide dismutase	104 88 90 91	0.9989 0.9988 0.9966 0.9926
*pOP-EA01332*	Predicted protein IFH-1 like	84 97 86 97	0.9951 0.9847 0.9986 0.9770
*GAPDH*	Glyceraldehyde 3-phosphate dehydrogenase	93 103 86 98	0.9997 0.9972 0.9976 0.9996
*NAD5*	NADH dehydrogenase subunit 5-like gene	92 98 90 86	0.9969 0.9994 0.9937 0.9935
*TUBULIN*	Alpha-tubulin 1	97 93 87 103	0.9981 0.9964 0.9992 0.9926
*UBIQUITIN*	Polyubiquitin	92 93 89 90	0.9979 0.9979 0.9999 0.9986
*ACTIN*	Actin	84 85 81 85	0.9996 0.9979 0.9988 0.9986

### Expression Levels of Candidate Reference Genes

**Figure 1:**
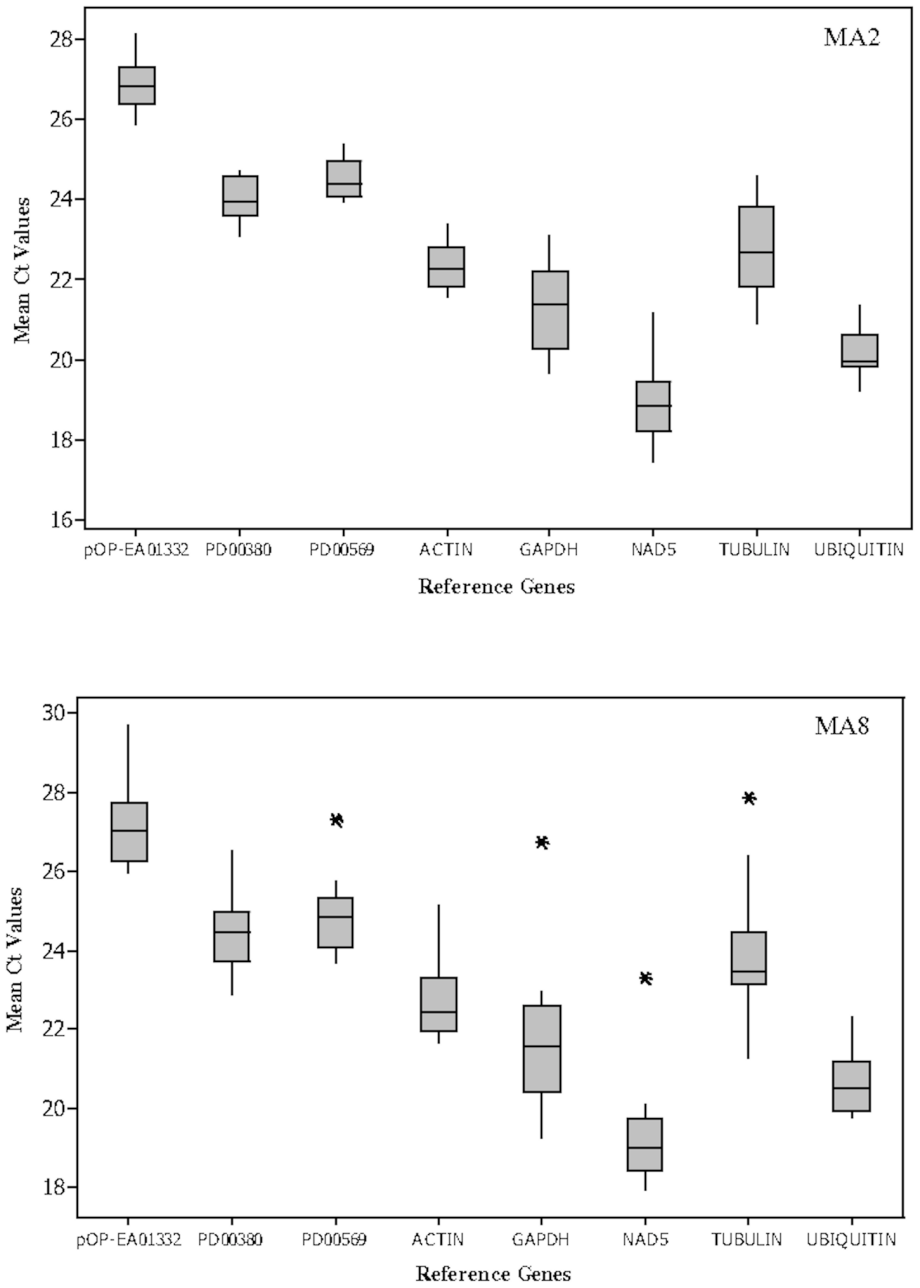
Mean Ct values of eight candidate reference genes across MA2 and MA8 tissue culture lines. The range of Ct values across the MA2 and MA8 tissue culture lines were exhibited in boxplot. Ct values of the candidate reference genes were widely distributed between 17 to 30 cycles. Lower and upper ends of the box represent 25th and 75 percentiles, respectively. Horizontal line inside the box is median. Whiskers below and above the box represent minimum and maximum values of the datasets, respectively. Asterisks represent the outliers.

Ct values derived from the amplification curve were used to measure the expression levels of candidate reference genes. As shown in Figure 1, the mean Ct values across MA2 and MA8 tissue culture lines were widely distributed between 17 to 30 cycles. Abundantly expressed genes across both tissue culture lines were transcripts coding for *NAD5* and *UBIQUITIN*, with mean Ct values in the range of 17 to 24 cycles. The *pOP-EA01332* gene exhibited the lowest expression levels compared to other candidate reference genes. Preliminary statistical analysis using the coefficient of variation (CV) was performed to determine the most stably expressed gene across this set of samples [41]. Lower CV values (1.90 to 4.10) calculated for the three novel reference genes showed that less variation in expression levels were observed across the tissue culture lines (Table S1). Classical reference genes such as *GAPDH*, *NAD5* and *TUBULIN* exhibited higher variation in their gene expression levels. The CV values for these genes were from 4.76 to 8.91 (Table S1). Therefore, a thorough analysis is required to shortlist the best combination of reference genes for an accurate and reliable normalization of gene expression data.

### Selection of Potential Reference Genes for Oil Palm Tissue Culture

Three Excel based tools, geNorm [19], NormFinder [38] and BestKeeper [39], were evaluated to select the most stably expressed reference genes across oil palm tissue culture lines. Expression stability of the candidate reference genes were firstly ranked using geNorm and the output was compared to the results from NormFinder and BestKeeper.

### GeNorm Analysis

**Figure 2:**
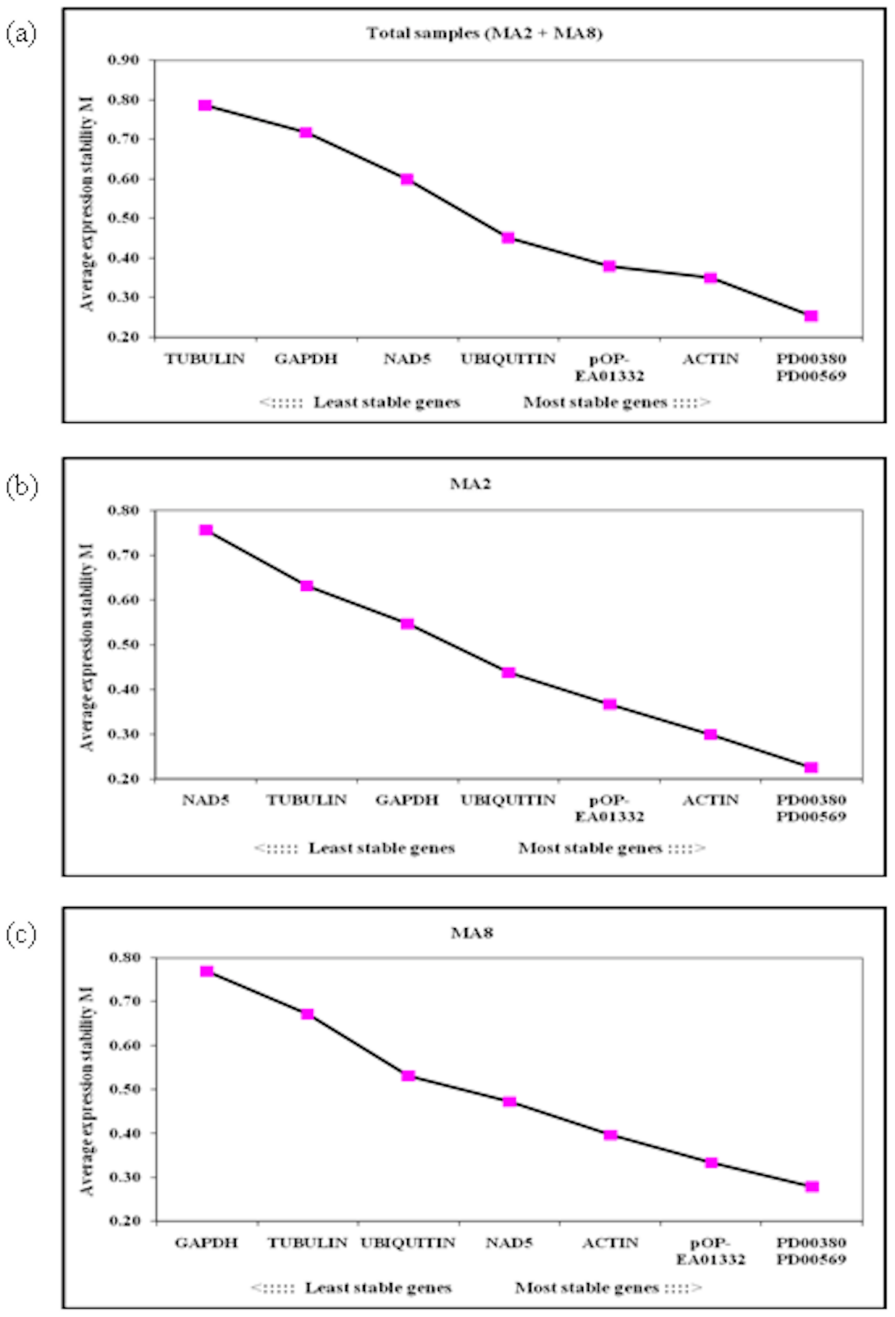
Determination of the most stably expressed reference genes across MA2 and MA8 tissue culture lines using geNorm software. Average expression stability values (*M*) were calculated for each reference gene. The least stable genes with higher *M* values were excluded in a stepwise manner until the two most stable reference genes were obtained for the tested tissue culture lines.

GeNorm was written by Vandesompele *et al*. [19] as a Visual Basic Application (VBA) for Microsoft Excel. In this program, gene expression stability measure *M* was calculated for each reference gene across the same set of samples. The least stable gene has a higher *M* value compared to the most stable gene. Elimination of the least stable gene was carried out in a stepwise manner until the two most stably expressed genes were obtained. Using the algorithm, the potential reference genes in this study were ranked based on their expression stability (Figure 2). In total samples that consisted of MA2 and MA8 non-normalized expression datasets, *PD00380* and *PD00569* were identified as the best-performing reference genes, while *TUBULIN* was the worst-scoring gene (Figure 2a). When the datasets were analysed separately as two individual tissue culture lines (Figure 2b and 2c), *PD00380* and *PD00569* still showed the most stable expression. The *NAD5* and *GAPDH* genes showed the least stable expression in the MA2 and MA8 tissue culture lines, respectively. Furthermore, the effect of the media treatments on the ranking of the reference genes was also investigated. Data in Figure S2 showed that *PD00380* and *PD00569* were still the most stably expressed reference genes across media T527 and T694. Among the classical housekeeping genes, *ACTIN* exhibited more stable expression. This gene was frequently ranked after *PD00380* and *PD00569* either across different tissue culture lines or media treatments.

**Figure 3:**
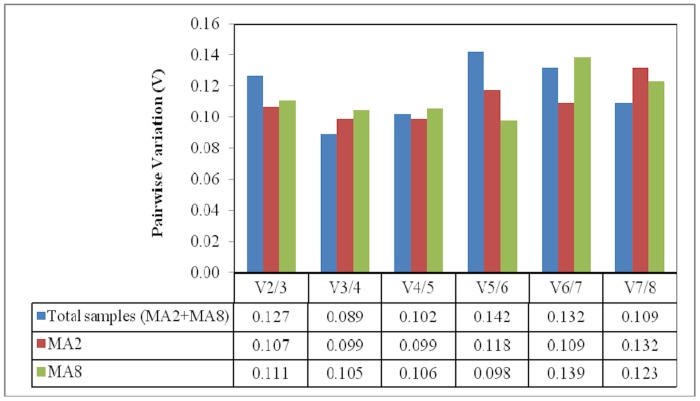
Optimal number of reference genes required for accurate and reliable normalization of gene expression data from each set of tissue culture lines as determined by geNorm software. Calculation of pairwise variation, V_n/n+1_ was performed between the two sequential normalization factors (NF_n_ and NF_n+1_) of reference genes across the same panel of samples. A cutoff value of 0.15 was used to determine whether the inclusion of an additional reference gene has a significant effect on the pairwise variation values.

In addition to ranking the genes according to *M* values, geNorm can also be used to determine the optimal number of reference genes required for accurate and reliable normalization of expression data across the tested samples. Pairwise variation, V_n/n+1_ was performed between the two sequential normalization factors (NF_n_ and NF_n+1_) of reference genes. In the event the value of V is lower than the recommended cutoff value of 0.15, addition of expression data from another reference gene is not required for calculation of a normalization factor based on the geometric mean [19]. As shown in Figure 3, the values of V_2+3_ for each set of data were less than 0.15. Therefore, only two reference genes, *PD00380* and *PD00569* are needed for accurate normalization of all the datasets in this study.

### NormFinder Analysis

NormFinder was also written as a VBA for Microsoft Excel. This application uses a mathematical model-based approach to estimate the expression variation of candidate reference genes [38] across the set of samples under investigation. The algorithm takes into consideration the intra- and intergroup variation for the calculation of an expression stability value. The most stably expressed gene has a lower expression stability value while the least stably expressed gene exhibits a higher expression stability value. Output from NormFinder analysis showed that *ACTIN, PD00380* and *PD00569* were the top three most stably expressed genes across all the tissue culture samples, MA2 and MA8 datasets (Table 4). Least stable genes for all the samples were predicted as *NAD5* and *GAPDH*. When the total samples were analyzed individually as MA2 and MA8 datasets, different combinations of least stably expressed genes were obtained. In the MA2 dataset, *TUBULIN* and *NAD5* were identified as the least stable genes. For the MA8 tissue culture line, *GAPDH* and *TUBULIN* were the least stable genes.

**Table 4:** Ranking of oil palm candidate reference genes using NormFinder analysis.

Rank	Total samples (MA2+MA8)	MA2	MA8
	Gene abbreviation Expression stability value	Gene abbreviation Expression stability value	Gene abbreviation Expression stability value
1	*ACTIN* 0.072	*ACTIN* 0.086	*ACTIN* 0.120
2	*PD00569* 0.109	*PD00380* 0.138	*PD00380* 0.135
3	*PD00380* 0.132	*PD00569* 0.158	*PD00569* 0.139
4	*pOP-EA01332* 0.150	*UBIQUITIN* 0.180	*pOP-EA01332* 0.144
5	*UBIQUITIN* 0.209	*pOP-EA01332* 0.192	*NAD5* 0.153
6	*TUBULIN* 0.220	*GAPDH* 0.348	*UBIQUITIN* 0.216
7	*GAPDH* 0.235	*NAD5* 0.404	*TUBULIN* 0.253
8	*NAD5* 0.245	*TUBULIN* 0.458	*GAPDH* 0.256
Best combination of two genes	*pOP-EA01332* and 0.065	*PD00380* and 0.103	*PD00380* and 0.080
	*PD00569*	*ACTIN*	*ACTIN*

NormFinder analysis also calculated the stability value for two reference genes that could be used in parallel for normalization purposes. The output on gene ranking from NormFinder was similar to geNorm. However, the combination of the two best reference genes was slightly different, which could be due to the different statistical algorithms applied in both applications. The best combination of the two genes for all the samples were *pOP-EA01332* and *PD00569* with a stability value of 0.065, while *PD00380* and *ACTIN* were identified as the most suitable genes for MA2 and MA8 (Table 4).

### BestKeeper Analysis

BestKeeper is an Excel based spreadsheet software application [39]. Average Ct values were used to calculate the coefficient of variance (CV) and SD for each of the reference genes. Genes with higher variation were classified as least stable whereas genes with lower variation were more stable. Based on this analysis, *PD00569* and *pOP-EA01332* were ranked as the most stably expressed genes across all the datasets with the CV ± SD values ranged from 1.66±0.41 to 2.95±0.80 (Table 5). Similarly the least stable genes, *GAPDH* and *TUBULIN* were observed in total samples and MA8 datasets. Expression levels of both genes were inconsistent across the tissue culture lines as the SD values were higher than 1. In the MA2 datasets, *NAD5* (4.29±0.81) followed by *GAPDH* (3.91±0.83) exhibited least stable expression levels. Grouping of reference genes according to expression stability were consistent with the output generated from geNorm and NormFinder. A distinct expression stability cluster was detected between novel and classical reference genes. The former was always grouped as the most stable cluster while the latter formed the least stable cluster.

**Table 5:** Ranking of oil palm candidate reference genes according to coefficient of variance (CV) and standard deviation (SD) using BestKeeper analysis.

Rank	Total samples (MA2+MA8)	MA2	MA8
	Gene abbreviation CV ± SD	Gene abbreviation CV ± SD	Gene abbreviation CV ± SD
1	*PD00569* 2.21±0.55	*PD00569* 1.66±0.41	*PD00569* 2.73±0.68
2	*pOP-EA01332* 2.47±0.67	*pOP-EA01332* 1.93±0.52	*pOP-EA01332* 2.95±0.80
3	*PD00380* 2.69±0.65	*PD00380* 2.03±0.49	*UBIQUITIN* 3.02±0.62
4	*ACTIN* 2.81±0.63	*ACTIN* 2.26±0.50	*PD00380* 3.06±0.75
5	*UBIQUITIN* 2.93±0.60	*UBIQUITIN* 2.48±0.50	*ACTIN* 3.30±0.75
6	*NAD5* 4.39±0.84	*TUBULIN* 3.70±0.84	*NAD5* 4.52±0.87
7	*TUBULIN* 4.60±1.07	*GAPDH* 3.91±0.83	*TUBULIN* 5.00±1.19
8	*GAPDH* 4.97±1.07	*NAD5* 4.29±0.81	*GAPDH* 6.20±1.34

The BestKeeper software also incorporated a pairwise correlation analysis among all possible pairings of the candidate reference genes and correlation analysis of Ct values from each candidate reference gene with the BestKeeper index or geometric mean. This index was calculated from Ct values generated by all of the candidate reference genes. Results from pairwise correlation analysis showed *PD00380* and *PD00569* as the most significantly correlated genes in total samples (Table S2), MA2 (Table S3) and MA8 (Table S4) datasets. The recommended gene-pair recorded the highest Pearson correlation coefficient (r) of 0.899 to 0.954 at the *p*-value of 0.001 across all datasets. The output was consistent with the pairwise variation analysis in geNorm. In addition, the BestKeeper index computed for total samples (Table S2) and MA8 (Table S4) were tightly correlated with the Ct values contributed by each of the reference genes. The r values were in the range of 0.757 to 0.968 with the majority of p-values computed as 0.001. As for the MA2 datasets, Ct values from *NAD5* were excluded from the calculation of the BestKeeper index as the reference genes exhibited higher variation (r = 0.490) across the tested samples (Table S3). Additional information from this analysis has enabled a robust selection of optimal reference genes for normalization of gene expression data.

### Validation of Potential Reference Genes

**Figure 4:**
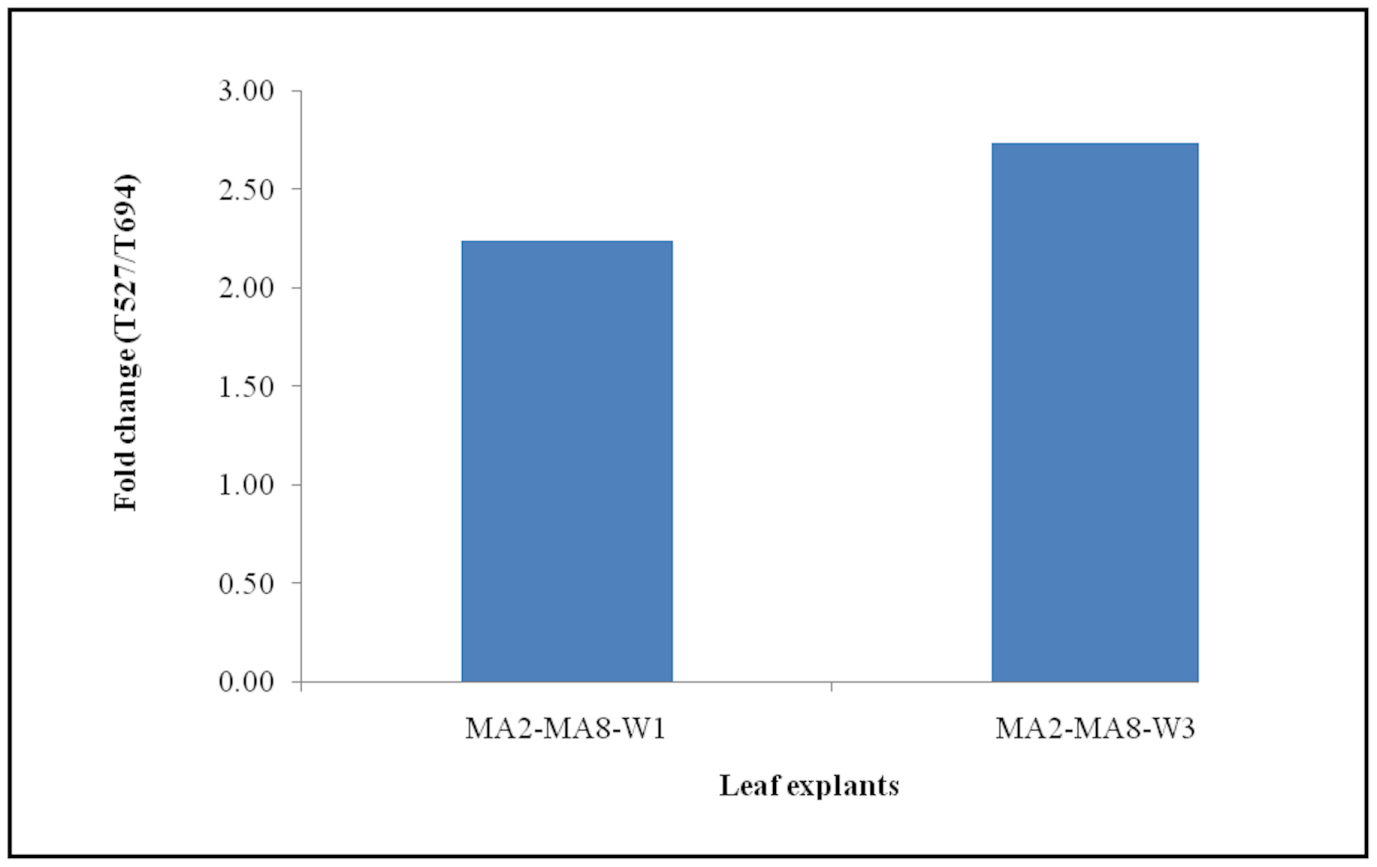
Fold change of PD00088 across MA2 and MA8 oil palm leaf explants as determined from the cDNA microarray data analysis. Microarray datasets from MA2 and MA8 tissue culture lines were analysed using statistical software known as Significance Analysis of Microarrays (SAM), which was developed by Tusher et al., 2001 and deposited as one of the packages under R for window. This software was used to identify differentially expressed genes at each time point between media T527 and T694. Two class unpaired data analysis was selected and the following parameters were applied for each analysis: False Discovery Rate (FDR) of zero, q-value of zero and gene expression ratio cutoff was fixed at 2-fold change.

**Figure 5:**
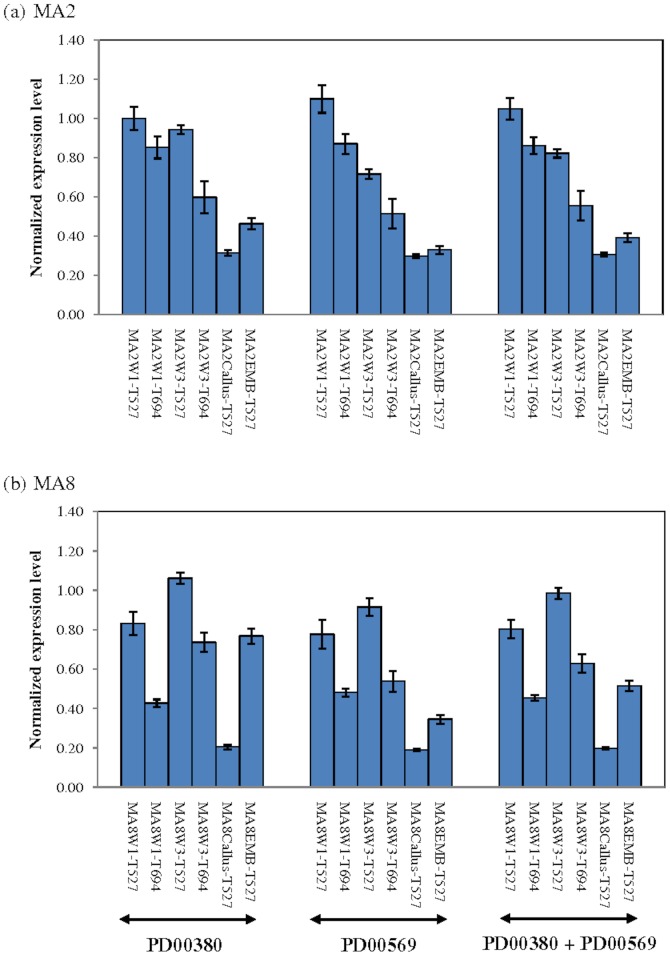
Expression profiling of PD00088 across oil palm leaf explants, callus and embryoids using RT-qPCR. Expression levels of PD00088 in leaf explants (W1, W3), callus and embryoids were normalized with PD000380, PD00569 or combination of both reference genes. Calculation of standard deviation on normalized gene expression level was done using geNorm v3.4 [19]. The error bars represent ± standard deviation (SD).

Based on the results from three independent analyses, *PD00380* and *PD00569* were selected as the most suitable reference genes for this study. Both genes were used singly or in combination to normalize the raw RT-qPCR data obtained from expression profiling of *PD00088* across Week_1 (W1) and Week_3 (W3) leaf explants from MA2 and MA8 tissue culture lines that were cultured on media T527 and T694. *PD00088* encodes for a putative ethylene-responsive transcription factor 3-like gene that contains a binding site for the AP2 DNA binding domain, which is postulated to be involved in somatic embryogenesis. A previous study using a cDNA microarray platform showed that the transcript for *PD00088* was highly expressed in W1 and W3 leaf explants from media T527 as compared to media T694 across MA2 and MA8 tissue culture lines (Figure 4). Reproducible expression patterns of PD00088 were observed in RT-qPCR when the expression data were normalized using either single or two reference genes (Figure 5). Slight differences in the expression levels were noticed in the data normalized with either *PD00380* or *PD00569*. However, the discrepancies were reduced with the usage of two reference genes at the same time, as this approach takes into consideration the geometric mean of two genes for calculation of the normalization factor. Similar outcomes were also obtained for normalized expression data from callus and embryoids (Figure 5). These results indicate the importance of using more than one reference gene for normalization of RT-qPCR expression data and the selected reference genes are deemed suitable for this study.

## Discussion

Research to unravel the complex molecular mechanisms underlying SE in oil palm has become extensive, resulting in an increasing amount of sequence and gene information in public databases. Expression patterns of these SE related genes have usually been assayed across various developmental stages of oil palm tissue culture as part of the effort to gauge their potential biological function. An in-depth understanding of the role of these genes will greatly assist in the identification of candidate expression markers for enhancement of the SE process.

RT-qPCR is one of the techniques that is commonly used to quantify the relative expression levels of the gene of interest in plants. However, due to the potential systematic variation introduced by total RNA, first strand cDNA synthesis and RT-qPCR assay [20], there is a need to normalize the raw expression data with constantly expressed internal controls for accurate and reliable results. In recent publications, two or more reference genes with validated expression stability have been used in the normalization of gene expression levels [28], [30], [41], [42], [43], [44]. Utilization of one single reference gene in normalization is no longer advisable as the latest findings show that no single reference gene is consistently expressed across all the tested plant tissues or experimental conditions [44], [45], [46].

For this study, three novel (*PD00380, PD00569, pOP-EA01332*) oil palm reference genes and five classical (*GAPDH, NAD5, TUBULIN, UBIQUITIN, ACTIN*) oil palm housekeeping genes were chosen for evaluation across tissue culture samples. Primer pairs for these genes were carefully designed to ensure amplification of specific PCR products from reverse transcribed cDNA. The presence of a single amplicon peak in the melting curve analysis (Figure S3) further confirmed the amplification of a specific PCR product [20], [47], [48]. Across all the available datasets (total samples, MA2 and MA8 tissue culture lines, different media treatments), *PD00380* and *PD00569* were ranked as the top two most stably expressed genes by geNorm (Figure 2, Figure S2). Results from Blast2GO analysis supported that both genes were categorized under different functional classes (Table 2). Therefore, the chance of geNorm in selecting co-regulated genes was reduced [19], [43]. Utilization of genes from different functional classes was also taken into consideration by Artico et al. [43] and Brunner et al. [49] in their search for reference genes with stable expression across various tissues of cotton and poplar, respectively. Output from NormFinder and BestKeeper analysis showed that *PD00380* and *PD00569* were still ranked amongst the top three genes with the most stable expression across oil palm tissue culture. The ranking position of these genes was slightly different from geNorm. Slight discrepancies of gene ranking among the three software are common due to the differences in the statistical algorithms and such observations have been reported recently in bamboo [28], eggplant [50] and tung tree [51].

The optimal number of reference genes varies in different experimental systems. In our case, geNorm analysis has recommended the geometric mean from two reference genes for calculation of the normalization factor (Figure 3). As the expression patterns from MA2 and MA8 tissue culture lines will be compared to each other, *PD00380* and *PD00569* with stable expression across all the datasets are the best choice of reference genes. The initial study by Vandesompele *et al*. [19] across 13 different human tissues had suggested a minimal usage of the three most stable reference genes. However, as more research has been carried out in plants, the decision on whether to use two or more reference genes has been based on the considerations of practicality [42] and research purposes [47], [52]. With this in mind, the normalization factor from two reference genes has been used for several plants such as *Platycladus orientalis* [41], cotton [43] and Chinese wolfberry [53].

Overall results showed that the expression stability of novel reference genes outperformed the classical housekeeping genes. Similar observations were reported previously for *Brassica juncea* [20] and soybean [44]. Since the novel genes were identified from the analysis of cDNA microarray data generated from tissue culture materials, such findings are not surprising. Advantages of mining candidate reference genes from publicly available microarray experiments has also been demonstrated for model plants such as Arabidopsis [21] and rice [24]. As for the classical housekeeping genes, their expression stability is dependent on plant species. Genes that frequently appeared to be least stable across our tested samples were *GAPDH* and *TUBULIN*. Good performance of *GAPDH* as a reference gene has been shown for citrus [27], Chinese wolfberry [53] and cotton under different stress conditions [54]. However its expression was unstable across papaya fruit samples [30]. Although *TUBULIN* is poorly ranked in bamboo [28] and peanut [52], it was deemed one of the most appropriate reference genes in banana [26] and across various developmental stages of somatic embryos in conifer species, *Pinus pinaster* and *Picea abies* [55]. Another classical housekeeping gene, *UBIQUITIN*, which performed poorly in our study, was also selected as one of the stably expressed gene in longan tree embryogenic cultures [56]. These outcomes again emphasize the importance of evaluating the expression stability of classical housekeeping genes before selecting them as an internal control for RT-qPCR.

The oil palm SE process in this study was initiated from leaf explants, which dedifferentiated into callus and subsequently somatic embryos. The presence of tissue culture samples from undifferentiated and differentiated phases across two auxin concentrations have made the selection of reference genes quite challenging as distinct groups of genes will be expressed. In order to increase the chances of selecting the most suitable reference genes, classical housekeeping genes were evaluated in parallel with novel reference genes. This approach also proved useful for longan SE and resulted in the recommendation of *UBIQUITIN* and iron superoxide dismutase (*FeSOD*) as the best combination of reference genes in the longan system [56]. However another class of SOD, designated as manganese superoxide dismutase (*MnSOD*) was classified as the least stable gene in longan SE as opposed to oil palm. Studies in *Nicotiana plumbaginifolia* revealed that the expression of *MnSOD* was induced in the plant cells during conditions of metabolic stress in tissue culture [57].

Hormones and stress are essential for the induction of SE through the tissue culture process. The hormone ethylene was found to be important for SE in *Medicago truncatula* [58] and *Hevea brasiliensis* [59]. In both plants, ethylene-responsive transcription factors from the AP2/ERF superfamily were responsible for promoting the SE process through the regulation of ethylene responsive genes. Expression of *MtSERF1* from *M. truncatula* was detected in the embryogenic calli and globular somatic embryo, while several AP2/ERF genes from *Hevea* were highly expressed in the calli from the embryogenic line. Oil palm *PD00088* (a putative ethylene-responsive transcription factor 3-like) belongs to this superfamily. Due to its potential substantive role in SE, this gene was selected to validate the applicability of *PD00380* and *PD00569* as reference genes. Results from RT-qPCR (Figure 5) are highly consistent with cDNA microarray data (Figure 4). Higher accumulation of this transcript in the somatic embryo is in agreement with the finding from *M. truncatula* [58]. This study showed that the geometric mean from two reference genes provide a better normalized expression levels as it is not sensitive to subtle changes. Thus, normalization with two reference genes are highly recommended.

## Conclusions

Systematic selection of the most stably expressed and the best combination of reference genes for RT-qPCR was established in oil palm tissue culture samples. Based on the analysis of three different statistical algorithms (geNorm, NormFinder, BestKeeper), *PD00380* and *PD00569* were selected as the most appropriate reference genes for accurate and reliable normalization of gene expression data from RT-qPCR of oil palm tissue culture samples. These genes outperformed the classical housekeeping genes and the geometric mean from two reference genes was sufficient to normalize the variations introduced in this study. The primer sequences of the eight candidate reference genes presented here would be valuable to the oil palm research community working on the expression profiling across other tissue culture samples or other oil palm tissues. By following the described method, identification of the most stably expressed reference genes for different sets of experiments can be done quickly. This will facilitate the functional characterization of genes associated with SE, yield, biotic and abiotic stresses.

## Materials and Methods

### Plant Materials

Tissue culture samples in this study were obtained from the EBOR Tissue Culture Laboratory (now Sime Darby Berhad, Malaysia). Leaf explants from two different palm trees of *tenera* fruit type [Deli *dura* x URT (Ulu Remis *Tenera*) *pisifera*) were used to generate two tissue culture lines, designated as MA2 and MA8. For each tissue culture line, the same source of leaf explant was placed on two different media treatments, T527 and T694. Tissue culture media T527, that consisted of 50 mg/l naphthalene acetic acid (NAA) and 0.5 g/l activated charcoal in the Murashige & Skoog (MS) basal culture medium [60], successfully produced embryogenic callus (EC) followed by embryoids (EMB). Whereas tissue culture media T694, that contained 5 mg/l NAA and 100 mg/l arginine in the MS basal culture medium, produced non-embryogenic callus (NEC). Leaf explants were collected before the start of the tissue culture process (Day_0), after Week_1 (W1), Week_2 (W2), Week_3 (W3), Week_4 (W4) and Week_8 (W8) on culture media, followed by sampling of the callus and EMB. A total of 14 and 12 tissue culture samples were collected from MA2 and MA8, respectively. In MA2 tissue culture line, the 14 samples comprised of 1 Day_0 leaf explants, 5 W1 to W8 leaf explants from media T527, 2 different stages of callus from media T527, 1 EMB from media T527 and 5 W1 to W8 leaf explants from media T694. For MA8 tissue culture line, 12 samples comprised of 1 Day_0 leaf explants, 5 W1 to W8 leaf explants from media T527, 1 callus from media T527 and 5 W1 to W8 leaf explants from media T694. All the samples were stored at -80°C prior to RNA extraction.

### Total RNA Extraction, Purification and Quality Assessment

Total RNA was extracted from various stages of tissue culture samples using the NTES (NaCl-Tris-EDTA-SDS) method with some minor modifications [61]. Total RNA was purified from DNA contamination using the RNeasy Mini Kit and RNase-free DNase I according to the manufacturer's instructions (Qiagen USA, Valencia, CA). The purity and quantity of the purified total RNA was determined using a NanoDrop ND-1000 UV-Vis Spectrophotometer (Thermo Fisher Scientific Inc.), and the integrity was assessed by electrophoretic fractionation on an Agilent 2100 Bioanalyzer and a RNA 6000 Nano LabChip (Agilent Technologies, CA).

### Primer Design

Eight potential reference genes were selected for evaluation across various developmental stages of oil palm tissue culture. Three novel reference genes, *PD00380*, *PD00569* and *pOP-EA01332* were identified from an oil palm cDNA microarray study across EC, NEC, EMB, ST, INF, kernel at 12 WAA, mesocarp at 15 WAA and roots from six months old seedling palms [37]. Another five genes were the classical housekeeping genes *GAPDH*, *NAD5*, *TUBULIN*, *UBIQUITIN* and *ACTIN*, which were selected based on a literature review [16]. Blast2GO analysis was performed across these genes for assignment of functional classification based on GO terms [40].

Gene-specific primers were designed to locate on either different exons or spanning the exon-exon junctions of the cDNA [47] to avoid the co-amplification of the genes from genomic DNA. Oil palm genomic sequences associated with the housekeeping genes were retrieved from MPOB's In-house Genomics Sequence Database. Alignment between the cDNAs and genomic sequences were performed using Spidey program from NCBI [62] to determine the putative exon-exon junctions of these genes. This information was then used to design gene-specific primers flanking the region of interest using Primer3 software [63]. Input parameters for primer design were as described: primer length (20 to 27 bases), primer GC content (40 to 60%), primer Tm (60 to 67°C) and amplicon length (100 to 150 bp). BLASTN search against the GenBank database was performed to confirm the specificity of each designed primer. The HPLC-purified primers were purchased from Bio Basic Canada Inc.

### Reverse Transcription Quantitative Real-time PCR

Synthesis of first-strand cDNA from 2 µg of total RNA samples from MA2 and MA8 was carried out using the High-capacity cDNA Reverse-Transcription Kit according to the manufacturer's instruction (Applied Biosystems). The first-strand cDNAs were used as templates in the SYBR Green based RT-qPCR using the Eppendorf Mastercycler ep realplex (Eppendorf, Germany). The 20 µl PCR reaction comprised of 4 µl cDNA template, 0.2 µM of reverse primer, 0.2 µM of forward primer and 1x KAPA SYBR FAST Universal 2X qPCR Master Mix (KAPA Biosystems). PCR was performed as follow: 95°C, 3 min for 1 cycle; 95°C, 3 sec and 60°C or 63°C (depending on the annealing temperature of primer pairs), 20 sec for 40 cycles and followed by a melting curve analysis at 60°C to 95°C with 0.4°C increase in temperature at each step. For each total RNA sample, a no reverse transcriptase control (NRT) was included as a control to determine whether the sample was freed from genomic DNA contamination. In addition, a non-template control (NTC) was also included as a negative control for each primer pair.

PCR amplification efficiencies and *R^2^* values of primers were determined across each pool of cDNA from MA2T527 (Day_0 leaf explants, W1 to W8 leaf explants, callus and EMB), MA2T694 (Day_0 leaf explants and W1 to W8 leaf explants), MA8T527 (Day_0 leaf explants, W1 to W8 leaf explants and callus) and MA8T694 (Day_0 leaf explants and W1 to W8 leaf explants). Ct values were measured across 5 different concentration of pooled cDNA (1, 2, 4, 8 and 16 ng) and the PCR amplification efficiencies were determined from the standard curves generated through the plotting of mean Ct values versus log10 cDNA concentration using the following calculation;

PCR amplification efficiencies, Ex  = [10^(-1/slope)^−1]×100%

(slope represents the slope of linear regression)

This was followed by the RT-qPCR of these primers across individual cDNA samples (10 ng) from MA2T527, MA2T694, MA8T527 and MA8T694.

### Data Analysis

The Ct values for each sample were retrieved using Realplex software version 2.2 (Eppendorf, Germany). Data analysis was carried out in Microsoft Excel. Average Ct values from three replicates were calculated and transformed into relative expression quantities using the delta Ct method, Ex∧(minCt - sampleCt). The most stable reference genes across the tissue culture samples were selected based on geNorm v3.4 [19], NormFinder v0.953 [38] and BestKeeper [39] software. Input data for geNorm and NormFinder are the relative expression quantities, while BestKeeper analysis is based on the average raw Ct values.

## Supporting Information

File S1 **List of top 75 gene clones identified from analysis of cDNA microarray datasets from tissue culture materials and mature tissues.**
(XLS)

File S2 **Determination of the most stably expressed genes across tissue culture materials and mature tissues using geNorm software.** Expression levels for each reference gene were measured across tissue culture materials (NEC, EC, EMB, ST, seven-day tissue culture explants) and mature tissues (LEAF, mesocarp, kernel, root and INF). Average expression stability values (M) was calculated for each reference gene. The least stable genes with higher M values were excluded in a stepwise manner until the most stable reference genes were shortlisted.
(DOC)

Figure S1 **Determination of PCR amplification efficiencies (Ex) and correlation coefficient (** ***R^2^*** **) values for** ***PD00569*** **using the slope of standard curve.** The estimated Ex for *PD00569* ranged from 88 to 104% and the *R^2^* were given as 0.9926 to 0.9989.
(DOC)

Figure S2 **Determination of the most stably expressed reference genes across media treatment T527 and T694 using geNorm software.** Average expression stability values (*M*) were calculated for each reference gene. The least stable genes with higher *M* values were excluded in a stepwise manner until the two most stable reference genes were obtained for the tested tissue culture media.
(DOC)

Figure S3 **Melting curve generated for** ***PD00569*** **across tissue culture samples collected from MA2 and MA8 tissue culture lines.** The presence of a single amplicon peak indicated the amplification of a specific PCR product.
(DOC)

Table S1 **Preliminary statistical analysis of oil palm candidate reference genes using the coefficient of variation (CV).**
(DOC)

Table S2 **Pair-wise correlation analysis and correlation analysis of oil palm candidate reference genes across the total samples (MA2 and MA8 tissue culture lines).**
(DOC)

Table S3 **Pair-wise correlation analysis and correlation analysis of oil palm candidate reference genes across the MA2 tissue culture line.**
(DOC)

Table S4 **Pair-wise correlation analysis and correlation analysis of oil palm candidate reference genes across the MA8 tissue culture line.**
(DOC)
