# Correction: Evaluation of Reference Genes for Quantitative Real-Time PCR in Oil Palm Elite Planting Materials Propagated by Tissue Culture

**DOI:** 10.1371/journal.pone.0110079

**Published:** 2014-10-01

**Authors:** The PLOS ONE Staff

There are errors in the paper. Please see below for descriptions of the errors and their corrections.

There are errors in the Funding section. The correct funding information is as follows: The research work was supported by Malaysian Palm Oil Board (MPOB) through research grant R000999000-RB01-J. The MPOB Director-General supported publication of the manuscript and did not influence the content.

An affiliation for the first author is missing. Pek-Lan Chan is affiliated with #1 Advanced Biotechnology and Breeding Centre, Malaysian Palm Oil Board (MPOB), No. 6, Persiaran Institusi, Bandar Baru Bangi, Kajang, Selangor, Malaysia, and #3 School of Biosciences and Biotechnology, Faculty of Science and Technology, Universiti Kebangsaan Malaysia, UKM Bangi, Selangor, Malaysia.

There are errors in Tables 3, 4, and 5. Please see the correct Tables here.

**Table 3:** PCR amplification efficiencies (Ex) and correlation coefficient (*R* ^2^) values of oil palm candidate reference genes.

Gene abbreviation	PCR amplification efficiencies, Ex (%)				Correlation coefficient *(R* ^2^)			
	MA2		MA8		MA2		MA8	
	T527	T694	T527	T694	T527	T694	T527	T694
*PD00380*	89	86	88	93	0.9987	0.9856	0.9976	0.9977
*PD00569*	104	88	90	91	0.9989	0.9988	0.9966	0.9926
*pOP-EA01332*	84	97	86	97	0.9951	0.9847	0.9986	0.9770
*GAPDH*	93	103	86	98	0.9997	0.9972	0.9976	0.9996
*NAD5*	92	98	90	86	0.9969	0.9994	0.9937	0.9935
*TUBULIN*	97	93	87	103	0.9981	0.9964	0.9992	0.9926
*UBIQUITIN*	92	93	89	90	0.9979	0.9979	0.9999	0.9986
*ACTIN*	84	85	81	85	0.9996	0.9979	0.9988	0.9986

**Table 4:** Ranking of oil palm candidate reference genes using NormFinder analysis.

Rank	Total samples (MA2+MA8)		MA2		MA8	
	Gene abbreviation	Expression stability value	Gene abbreviation	Expression stability value	Gene abbreviation	Expression stability value
1	*ACTIN*	0.072	*ACTIN*	0.086	*ACTIN*	0.120
2	*PD00569*	0.109	*PD00380*	0.138	*PD00380*	0.135
3	*PD00380*	0.132	*PD00569*	0.158	*PD00569*	0.139
4	*pOP-EA01332*	0.150	*UBIQUITIN*	0.180	*pOP-EA01332*	0.144
5	*UBIQUITIN*	0.209	*pOP-EA01332*	0.192	*NAD5*	0.153
6	*TUBULIN*	0.220	*GAPDH*	0.348	*UBIQUITIN*	0.216
7	*GAPDH*	0.235	*NAD5*	0.404	*TUBULIN*	0.253
8	*NAD5*	0.245	*TUBULIN*	0.458	*GAPDH*	0.256
Best combination of two genes	*pOP-EA01332* and *PD00569*	0.065	*PD00380* and *ACTIN*	0.103	*PD00380* and *ACTIN*	0.080

**Table 5:** Ranking of oil palm candidate reference genes according to coefficient of variance (CV) and standard deviation (SD) using BestKeeper analysis.

Rank	Total samples (MA2+MA8)		MA2		MA8	
	Gene abbreviation	CV±SD	Gene abbreviation	CV±SD	Gene abbreviation	CV±SD
1	*PD00569*	2.21±0.55	*PD00569*	1.66±0.41	*PD00569*	2.73±0.68
2	*pOP-EA01332*	2.47±0.67	*pOP-EA01332*	1.93±0.52	*pOP-EA01332*	2.95±0.80
3	*PD00380*	2.69±0.65	*PD00380*	2.03±0.49	*UBIQUITIN*	3.02±0.62
4	*ACTIN*	2.81±0.63	*ACTIN*	2.26±0.50	*PD00380*	3.06±0.75
5	*UBIQUITIN*	2.93±0.60	*UBIQUITIN*	2.48±0.50	*ACTIN*	3.30±0.75
6	*NAD5*	4.39±0.84	*TUBULIN*	3.70±0.84	*NAD5*	4.52±0.87
7	*TUBULIN*	4.60±1.07	*GAPDH*	3.91±0.83	*TUBULIN*	5.00±1.19
8	*GAPDH*	4.97±1.07	*NAD5*	4.29±0.81	*GAPDH*	6.20±1.34
