# Supplementary material for: Evaluation of Reference Genes for Quantitative Real-Time PCR in Oil Palm Elite Planting Materials Propagated by Tissue Culture
Source: PLoS One. 2014 Jun 13;9(6):e99774. doi: 10.1371/journal.pone.0099774 (PMC4057393; doi:10.1371/journal.pone.0099774)
Supplement: File S2 — Determination of the most stably expressed genes across tissue culture materials and mature tissues using geNorm software. Expression levels for each reference gene were measured across tissue culture materials (NEC, EC, EMB, ST, seven-day tissue culture explants) and mature tissues (LEAF, mesocarp, kernel, root and INF). Average expression stability values (M) was calculated for each reference gene. The least stable genes with higher M values were excluded in a stepwise manner until the most stable reference genes were shortlisted. (DOC) [file pone.0099774.s002.doc]

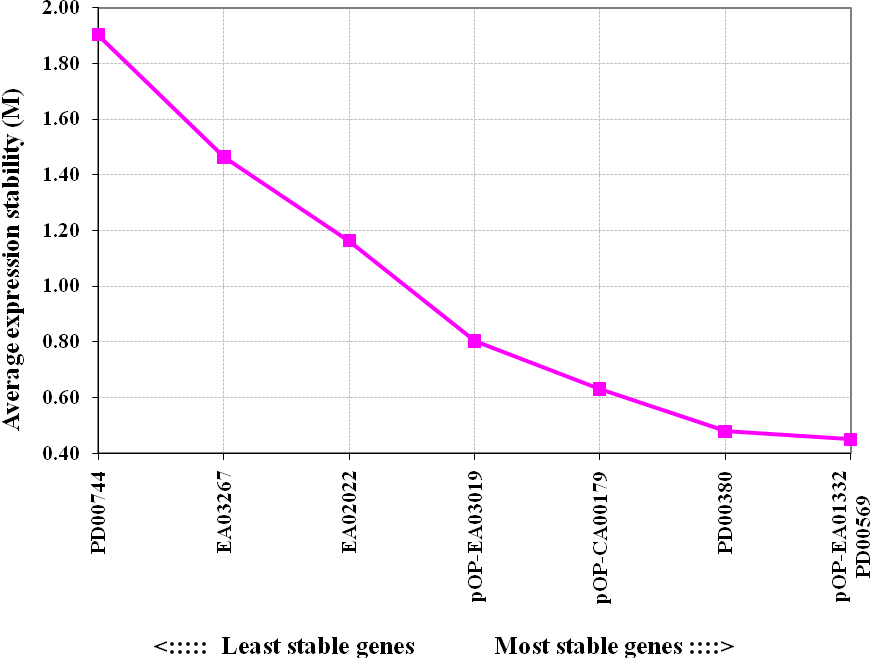


# File S2. Determination of the most stably expressed genes across tissue culture materials and mature tissues using geNorm software. Expression levels for each reference gene were measured across tissue culture materials (NEC, EC, EMB, ST, seven-day tissue culture explants) and mature tissues (LEAF, mesocarp, kernel, root and INF). Average expression stability values (M) was calculated for each reference gene. The least stable genes with higher M values were excluded in a stepwise manner until the most stable reference genes were shortlisted.
