# Supplementary material for: Evaluation of Reference Genes for Quantitative Real-Time PCR in Oil Palm Elite Planting Materials Propagated by Tissue Culture
Source: PLoS One. 2014 Jun 13;9(6):e99774. doi: 10.1371/journal.pone.0099774 (PMC4057393; doi:10.1371/journal.pone.0099774)
Supplement: Figure S1 — Determination of PCR amplification efficiencies (Ex) and correlation coefficient ( R2 ) values for PD00569 using the slope of standard curve. The estimated Ex for PD00569 ranged from 88 to 104% and the R2 were given as 0.9926 to 0.9989. (DOC) [file pone.0099774.s003.doc]

#
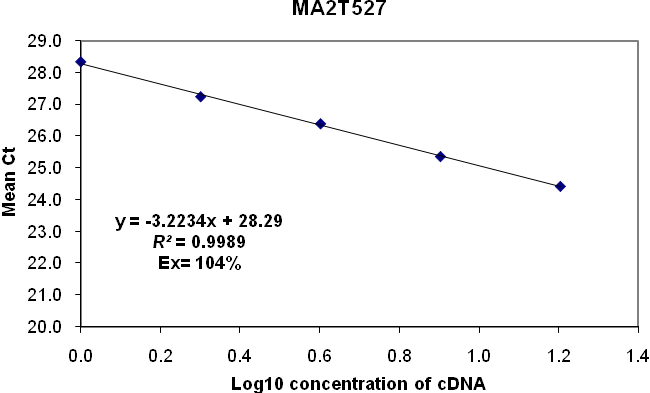

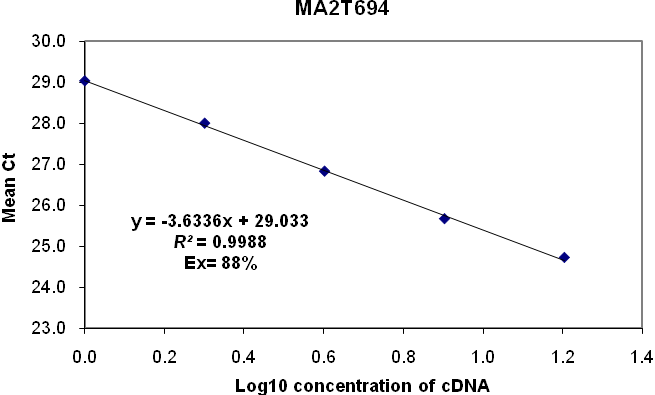


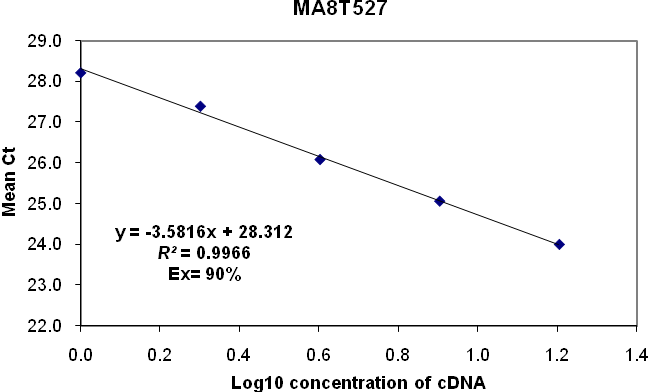

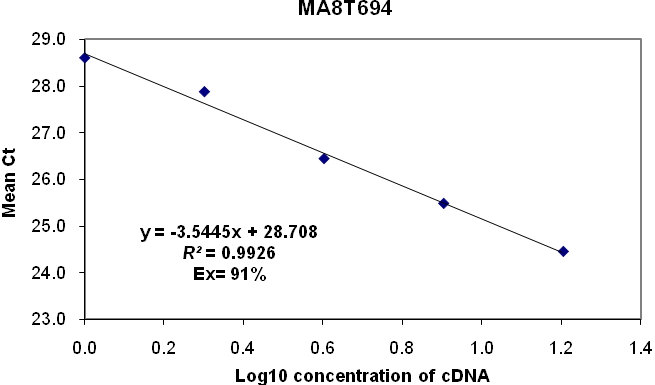


# Figure S1. Determination of PCR amplification efficiencies (Ex) and correlation coefficient (*R^2^*) values for *PD00569* using the slope of standard curve. The estimated Ex for *PD00569* ranged from 88 to 104% and the *R^2^* was given as 0.9926 to 0.9989.
