# Supplementary material for: Evaluation of Reference Genes for Quantitative Real-Time PCR in Oil Palm Elite Planting Materials Propagated by Tissue Culture
Source: PLoS One. 2014 Jun 13;9(6):e99774. doi: 10.1371/journal.pone.0099774 (PMC4057393; doi:10.1371/journal.pone.0099774)
Supplement: Figure S2 — Determination of the most stably expressed reference genes across media treatment T527 and T694 using geNorm software. Average expression stability values (M) were calculated for each reference gene. The least stable genes with higher M values were excluded in a stepwise manner until the two most stable reference genes were obtained for the tested tissue culture media. (DOC) [file pone.0099774.s004.doc]

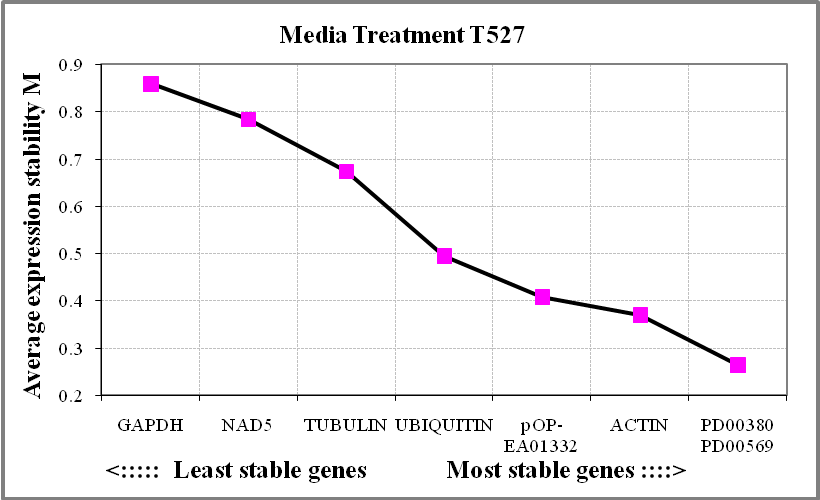


(a)


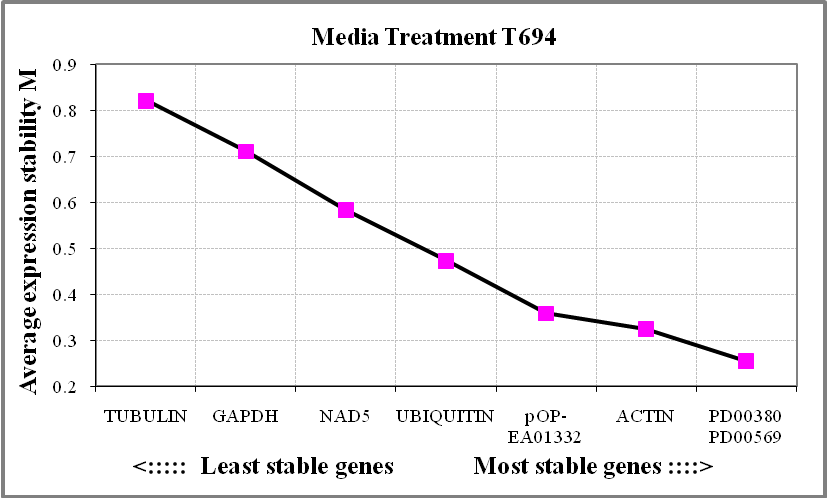


(b)

## Figure S2. Determination of the most stably expressed reference genes across media treatment T527 and T694 using geNorm software.

Average expression stability values (*M*) were calculated for each reference gene. The least stable genes with higher *M* values were excluded in a stepwise manner until the two most stable reference genes were obtained for the tested tissue culture media.
