# Supplementary material for: Evaluation of Reference Genes for Quantitative Real-Time PCR in Oil Palm Elite Planting Materials Propagated by Tissue Culture
Source: PLoS One. 2014 Jun 13;9(6):e99774. doi: 10.1371/journal.pone.0099774 (PMC4057393; doi:10.1371/journal.pone.0099774)
Supplement: Figure S3 — Melting curve generated for PD00569 across tissue culture samples collected from MA2 and MA8 tissue culture lines. The presence of a single amplicon peak indicated the amplification of a specific PCR product. (DOC) [file pone.0099774.s005.doc]

1. **MA2**

1. **MA8**

# Figure S3. Melting curve generated for *PD00569* across tissue culture samples collected from MA2 and MA8 tissue culture lines. The presence of a single amplicon peak indicated the amplification of a specific PCR product.
