# Supplementary material for: Evaluation of Reference Genes for Quantitative Real-Time PCR in Oil Palm Elite Planting Materials Propagated by Tissue Culture
Source: PLoS One. 2014 Jun 13;9(6):e99774. doi: 10.1371/journal.pone.0099774 (PMC4057393; doi:10.1371/journal.pone.0099774)
Supplement: Table S1 — Preliminary statistical analysis of oil palm candidate reference genes using the coefficient of variation (CV). (DOC) [file pone.0099774.s006.doc]

**Table S1. Preliminary statistical analysis of oil palm candidate reference genes using the coefficient of variation (CV).**

| Tissue culture line | *pOP-EA01332* | *PD00380* | *PD00569* | *ACTIN* | *GAPDH* | *NAD5* | *TUBULIN* | *UBIQUITIN* |
| --- | --- | --- | --- | --- | --- | --- | --- | --- |
| MA2 | 2.61 | 2.36 | 1.90 | 2.72 | 4.86 | 5.70 | 4.76 | 3.00 |
| MA8 | 3.93 | 4.10 | 3.89 | 4.36 | 8.91 | 7.11 | 7.23 | 3.78 |
