# Supplementary material for: Evaluation of Reference Genes for Quantitative Real-Time PCR in Oil Palm Elite Planting Materials Propagated by Tissue Culture
Source: PLoS One. 2014 Jun 13;9(6):e99774. doi: 10.1371/journal.pone.0099774 (PMC4057393; doi:10.1371/journal.pone.0099774)
Supplement: Table S2 — Pair-wise correlation analysis and correlation analysis of oil palm candidate reference genes across the total samples (MA2 and MA8 tissue culture lines). (DOC) [file pone.0099774.s007.doc]

**Table S2. Pair-wise correlation analysis and correlation analysis of oil palm candidate reference genes across the total samples (MA2 and MA8 tissue culture lines).**

|  | *pOP-EA01332* | *PD00380* | *PD00569* | *ACTIN* | *UBIQUITIN* | *GAPDH* | *NAD5* | *TUBULIN* |
| --- | --- | --- | --- | --- | --- | --- | --- | --- |
| vs. | HKG 1 | HKG 2 | HKG 3 | HKG 4 | HKG 5 | HKG 6 | HKG 7 | HKG 8 |
| HKG 2 | 0.858 | - | - | - | - | - | - | - |
| p-value | 0.001 | - | - | - | - | - | - | - |
| HKG 3 | 0.880 | 0.941 | - | - | - | - | - | - |
| p-value | 0.001 | 0.001 | - | - | - | - | - | - |
| HKG 4 | 0.863 | 0.811 | 0.881 | - | - | - | - | - |
| p-value | 0.001 | 0.001 | 0.001 | - | - | - | - | - |
| HKG 5 | 0.632 | 0.693 | 0.724 | 0.741 | - | - | - | - |
| p-value | 0.001 | 0.000 | 0.001 | 0.001 | - | - | - | - |
| HKG 6 | 0.745 | 0.794 | 0.794 | 0.838 | 0.709 | - | - | - |
| p-value | 0.001 | 0.001 | 0.001 | 0.001 | 0.001 | - | - | - |
| HKG 7 | 0.566 | 0.730 | 0.780 | 0.655 | 0.470 | 0.664 | - | - |
| p-value | 0.002 | 0.001 | 0.001 | 0.001 | 0.011 | 0.001 | - | - |
| HKG 8 | 0.725 | 0.795 | 0.779 | 0.844 | 0.575 | 0.864 | 0.661 | - |
| p-value | 0.001 | 0.001 | 0.001 | 0.001 | 0.001 | 0.001 | 0.001 | - |
|  |  |  |  |  |  |  |  |  |
| BestKeeper vs. | HKG 1 | HKG 2 | HKG 3 | HKG 4 | HKG 5 | HKG 6 | HKG 7 | HKG 8 |
| coeff. of corr. [r] | 0.859 | 0.922 | 0.941 | 0.929 | 0.757 | 0.927 | 0.803 | 0.907 |
| p-value | 0.001 | 0.001 | 0.001 | 0.001 | 0.001 | 0.001 | 0.001 | 0.001 |
