# Supplementary material for: Evaluation of Reference Genes for Quantitative Real-Time PCR in Oil Palm Elite Planting Materials Propagated by Tissue Culture
Source: PLoS One. 2014 Jun 13;9(6):e99774. doi: 10.1371/journal.pone.0099774 (PMC4057393; doi:10.1371/journal.pone.0099774)
Supplement: Table S3 — Pair-wise correlation analysis and correlation analysis of oil palm candidate reference genes across the MA2 tissue culture line. (DOC) [file pone.0099774.s008.doc]

**Table S3. Pair-wise correlation analysis and correlation analysis of oil palm candidate reference genes across the MA2 tissue culture line.**

|  | *pOP-EA01332* | *PD00380* | *PD00569* | *ACTIN* | *UBIQUITIN* | *GAPDH* | *NAD5* | *TUBULIN* |
| --- | --- | --- | --- | --- | --- | --- | --- | --- |
| vs. | HKG 1 | HKG 2 | HKG 3 | HKG 4 | HKG 5 | HKG 6 | HKG 7 | HKG 8 |
| HKG 2 | 0.734 | - | - | - | - | - | - | - |
| p-value | 0.002 | - | - | - | - | - | - | - |
| HKG 3 | 0.724 | 0.899 | - | - | - | - | - | - |
| p-value | 0.002 | 0.001 | - | - | - | - | - | - |
| HKG 4 | 0.781 | 0.728 | 0.851 | - | - | - | - | - |
| p-value | 0.001 | 0.002 | 0.001 | - | - | - | - | - |
| HKG 5 | 0.533 | 0.423 | 0.530 | 0.617 | - | - | - | - |
| p-value | 0.041 | 0.117 | 0.042 | 0.014 | - | - | - | - |
| HKG 6 | 0.537 | 0.669 | 0.683 | 0.731 | 0.795 | - | - | - |
| p-value | 0.039 | 0.006 | 0.005 | 0.002 | 0.001 | - | - | - |
| HKG 7 | -0.033 | 0.472 | 0.519 | 0.210 | 0.085 | 0.127 | - | - |
| p-value | 0.906 | 0.076 | 0.047 | 0.455 | 0.761 | 0.653 | - | - |
| HKG 8 | 0.467 | 0.674 | 0.673 | 0.686 | 0.356 | 0.740 | 0.245 | - |
| p-value | 0.080 | 0.006 | 0.006 | 0.005 | 0.194 | 0.002 | 0.379 | - |
| BestKeeper vs. | HKG 1 | HKG 2 | HKG 3 | HKG 4 | HKG 5 | HKG 6 | HKG 7 | HKG 8 |
| coeff. of corr. [r] | 0.679 | 0.883 | 0.927 | 0.864 | 0.679 | 0.856 | 0.490 | 0.809 |
| p-value | 0.005 | 0.001 | 0.001 | 0.001 | 0.005 | 0.001 | 0.063 | 0.001 |
