# Supplementary material for: Evaluation of Reference Genes for Quantitative Real-Time PCR in Oil Palm Elite Planting Materials Propagated by Tissue Culture
Source: PLoS One. 2014 Jun 13;9(6):e99774. doi: 10.1371/journal.pone.0099774 (PMC4057393; doi:10.1371/journal.pone.0099774)
Supplement: Table S4 — Pair-wise correlation analysis and correlation analysis of oil palm candidate reference genes across the MA8 tissue culture line. (DOC) [file pone.0099774.s009.doc]

**Table S4. Pair-wise correlation analysis and correlation analysis of oil palm candidate reference genes across the MA8 tissue culture line.**

|  | *pOP-EA01332* | *PD00380* | *PD00569* | *ACTIN* | *UBIQUITIN* | *GAPDH* | *NAD5* | *TUBULIN* |
| --- | --- | --- | --- | --- | --- | --- | --- | --- |
| vs. | HKG 1 | HKG 2 | HKG 3 | HKG 4 | HKG 5 | HKG 6 | HKG 7 | HKG 8 |
| HKG 2 | 0.918 | - | - | - | - | - | - | - |
| p-value | 0.001 | - | - | - | - | - | - | - |
| HKG 3 | 0.947 | 0.954 | - | - | - | - | - | - |
| p-value | 0.001 | 0.001 | - | - | - | - | - | - |
| HKG 4 | 0.901 | 0.828 | 0.890 | - | - | - | - | - |
| p-value | 0.001 | 0.001 | 0.001 | - | - | - | - | - |
| HKG 5 | 0.686 | 0.796 | 0.813 | 0.788 | - | - | - | - |
| p-value | 0.010 | 0.001 | 0.001 | 0.001 | - | - | - | - |
| HKG 6 | 0.827 | 0.837 | 0.823 | 0.879 | 0.685 | - | - | - |
| p-value | 0.001 | 0.001 | 0.001 | 0.001 | 0.010 | - | - | - |
| HKG 7 | 0.914 | 0.867 | 0.915 | 0.894 | 0.706 | 0.932 | - | - |
| p-value | 0.001 | 0.001 | 0.001 | 0.001 | 0.007 | 0.001 | - | - |
| HKG 8 | 0.860 | 0.820 | 0.814 | 0.908 | 0.604 | 0.943 | 0.901 | - |
| p-value | 0.001 | 0.001 | 0.001 | 0.001 | 0.029 | 0.001 | 0.001 | - |
|  |  |  |  |  |  |  |  |  |
| BestKeeper vs. | HKG 1 | HKG 2 | HKG 3 | HKG 4 | HKG 5 | HKG 6 | HKG 7 | HKG 8 |
| coeff. of corr. [r] | 0.938 | 0.928 | 0.942 | 0.951 | 0.783 | 0.956 | 0.968 | 0.945 |
| p-value | 0.001 | 0.001 | 0.001 | 0.001 | 0.002 | 0.001 | 0.001 | 0.001 |
